# The effects of yam gruel on lowering fasted blood glucose in T2DM rats

**DOI:** 10.1515/biol-2020-0096

**Published:** 2020-10-14

**Authors:** Xinjun Lin, Zongting Luo, Shuqin Pang, Carol Chunfeng Wang, Li Ge, Yanling Dai, Jian Zhou, Fang Chen, Xuepei Hong, Jiahui Zhang

**Affiliations:** Institute of Integrated Chinese and Western Medicine, Fujian University of Traditional Chinese Medicine, Fuzhou, 350122, Fujian, China; School of Nursing, Fujian University of Traditional Chinese Medicine, Fuzhou, 350122, Fujian, China; School of Nursing and Midwifery, Edith Cowan University, Perth, WA, 6027, Australia

**Keywords:** yam gruel, gut microbiota, short-chain fatty acids, 16S rRNA gene sequencing, supplement therapy

## Abstract

There is increasing evidence of the linkage between type 2 diabetes mellitus (T2DM) and gut microbiota. Based on our previous studies, we investigated the hypoglycemic mechanisms of yam gruel to provide a scientific basis for its popularization and application. Wistar rats were randomly divided into control and T2DM model groups. Rats in the model group were stimulated by a high-sugar/high-fat diet combined with an intraperitoneal injection of streptozotocin to induce T2DM. The T2DM rats were further subdivided randomly into three groups: (1) DM, (2) DM + yam gruel, and (3) DM + metformin. After 4 weeks of intervention, the changes in gut microbiota, short-chain fatty acids (SCFAs) (acetic acid, propionic acid, and butyric acid), the expression of G protein-coupled receptor 43 (GPR43), glucagon-like peptide-1 (GLP-1), peptide YY (PYY), and fasted blood glucose (FBG) levels were observed. Yam gruel intervention elevated the abundance of probiotic bacteria and increased the expression of SCFAs, GPR43 receptor, GLP-1, and PYY. It also reduced FBG levels. We conclude that yam gruel can lower FBG by promoting the growth of probiotic bacteria, increasing the content of SCFAs, and enhancing the expression of GPR43 receptor to increase the content of GLP-1 and PYY in serum.

## Abbreviations

T2DM : type 2 diabetes mellitus
STZ : streptozotocin
SCFAs : short-chain fatty acids
GPR43 : G protein-coupled receptor 43
GLP-1 : glucagon-like peptide-1
PYY : peptide YY
FBG : fasted blood glucose

## 1 Introduction

Diabetes mellitus is one of the most critical chronic noncommunicable diseases, threatening human health around the world. According to the statistics of the International Diabetes Federation, the number of diabetes patients worldwide reached 463 million in 2019 and is expected to rise to 578 million by 2030. Approximately 116 million people are living with diabetes mellitus in China, among whom type 2 diabetes mellitus (T2DM) accounts for more than 90% [1,2]. The morbidity of T2DM remains high, with numerous and severe complications that place heavy economic and care burdens on families and society [3,4,5].

With advances in research, the gut microbiota has increasingly become a new target for the prevention and treatment of T2DM [6]. In T2DM patients, the abundance of probiotics such as Bifidobacterium in the intestinal tract has been shown to decrease while the level of opportunistic pathogens such as Bacteroides increased significantly [7,8]. Furthermore, recent studies have reported that the abundance of probiotic bacteria can substantially influence the level of short-chain fatty acids (SCFAs), such as acetic acid, propionic acid, and butyric acid. Streptococcus [9], Oscillospira [10], and Phascolarctobacterium [11] are well-known SCFA-producing bacteria, the decrease of which could lead to a reduction in SCFAs. SCFAs are a regulatory signal of metabolism that can directly activate G protein-coupled receptor 43 (GPR43), promote the secretion of glucagon-like peptide-1 (GLP-1) and peptide YY (PYY), improve fasting hyperglycemia, and delay the development of diabetes [12,13].

It is well accepted that a sensible diet can be a primary factor of gut microbiota structure as well as an essential intervention for diabetes. Dietary nursing in traditional Chinese medicine is a special nutritional intervention that can be used as both food and medicine. Yam gruel is a classic food therapy documented in the *Records of Traditional Chinese and Western Medicine in Combination* written by Zhang [14]. A previous study by our group showed that yam gruel can facilitate the growth of Bifidobacterium and help lower blood glucose levels [15,16]. However, the specific hypoglycemic mechanism of yam gruel is unclear.

The current study aimed to determine the effects of yam gruel on the gut microbiota and SCFAs and to evaluate other vital indicators, including the GPR43 receptor, GLP-1, PYY, and fasted blood glucose (FBG). Ultimately, we believe that this work provides novel insight into the hypoglycemic mechanism of yam gruel and insight into T2DM prevention, providing a scientific basis for the popularization and application of yam gruel.

## 2 Materials and methods

### 2.1 Preparation of yam gruel

Raw *Dioscorea polystachya*, common name Chinese yam or “Huai Qing Fu” yam, was ordered and confirmed for its originality by a pharmacologist. The desired *Dioscorea polystachya* was cylindrical in shape of at least 2 cm in diameter with much mucus. The raw *Dioscorea polystachya* used in the study was purchased and immediately stored in the refrigerator. Following the classic recipe described in the *Records of Traditional Chinese and Western Medicine in Combination* [14], and the dosage used in our previous study [15], yam gruel was prepared every morning. The specific method was as follows: raw *Dioscorea polystachya* was peeled and sliced (125 g), mixed with 50 mL of water in a cooker and beaten into a paste. Cold water (250 mL) was added, and the yams were boiled over medium heat for 30 s. The mixture was boiled again for 30 s over low heat, and then the heat was turned off for 30 s. The procedure was repeated three times. During the processing, continual stirring is important to avoid yam sticking to the bottom of the cooker. Finally, the yam gruel was boiled in approximately 250 mL aliquots, which were about 0.5 g of yam per 1 mL, and then cooled to 37°C prior to use.

### 2.2 Animals and diet

Six-week-old male Wistar rats (150 ± 10 g) were purchased from Shanghai Slack Laboratory Animal Co., Ltd (Shanghai, China). All rats were housed in cages at a temperature between 25 ± 2°C and 55 + 5% humidity, maintaining a 12-h light/dark cycle (light on 08:00–20:00). The rats were given free access to food and water. After 1 week of adaptive feeding, 38 rats were randomly divided into a normal control (NC) group (*n* = 8) fed with a standardized diet, and a model group (*n* = 30) fed with a high-sugar/high-fat diet. The standardized diet was provided by the Animal Experiment Centre of Fujian University of Traditional Chinese Medicine, while the high-sugar/high-fat diet (10.0% lard, 15.0% sucrose, 4% cholesterol, 10% yolk powder, 0.3% cholate, and 60.7% standard diet) was provided by Minhou Wushi Experimental Animal Trading Co., Ltd (Fuzhou, China). After 6 weeks, all rats were fasted for 12 h. The rats in the model group were intraperitoneally injected with 1% streptozotocin (STZ) solution twice at a dose of 25 mg/kg (interval 2 days) while the NC group received an intraperitoneal injection of citric acid buffer at the same dose. After 1 week of observation, blood samples were collected from the tail vein once a day for two consecutive days and FBG ≥ 11.1 mmol/L was determined as a standard for T2DM rat [17]. T2DM rats were randomly divided into three subgroups: (1) DM (normal saline, *n* = 8), (2) DM + yam gruel (yam gruel, *n* = 7), and (3) DM + metformin (metformin, as a positive control, 100 mg/kg/day, *n* = 8). All interventions were delivered in a liquid state via a gavage needle, and the dose of gavage was set to be 15 mL/kg/day. Rats were fed with a standardized diet throughout the intervention. After 4 weeks of intervention, the rats were anaesthetized with pentobarbital (35 mg/kg, i.p.) and killed by cervical dislocation. Subsequently, blood from the abdominal aorta and stool from the colon were collected. Colon tissues were obtained rapidly, washed with ice cold phosphate-buffered saline, and then stored at −80°C.


**Ethical approval:** The research related to animal use has been complied with all the relevant national regulations and institutional policies for the care and use of animals and has been approved by the Animal Experiment Committee of the Fujian University of Traditional Chinese Medicine (2019-015).

### 2.3 Measurement of gut microbiota

Gut microbiota was detected by 16S rRNA gene sequencing technology. Microbial DNA was extracted from each sample using the QIAamp®DNA Stool Mini Kit (Hilden, Germany) according to the manufacturer’s instructions. DNA concentrations were determined using a Qubit quantification system (Thermo Scientific, Wilmington, DE, USA). The microbial V3-V4 hypervariable region of the 16S rRNA gene was amplified from genomic DNA using the primers 341 F (CCTACGGGNGGCWGCAG) and 805 R (GACTACHVGGGTATCTAATCC). Amplification was performed in a reaction mixture consisting of 1X KAPA HiFi Hot start Ready Mix, 0.1 µM primer 341 F, 0.1 µM primer 805 R, and 12.5 ng template DNA, giving a total volume of 50 µL per sample. Reactions were run in a T100PCR thermocycler (BIO-RAD) according to the following cycling program: 3 min of denaturation at 94°C, followed by 18 cycles of 30 s at 94°C (denaturation), 30 s at 55°C (annealing), and 30 s at 72°C (elongation), with a final extension at 72°C for 5 min. Subsequently, the amplified products were checked by 2% agarose gel electrophoresis and ethidium bromide staining. Amplicons were quantified using the Qubit quantification system and purified with Agencourt AMPure XP Beads (Beckman Coulter Genomics, MA, USA). The concentration of the pooled libraries were determined using the Qubit quantification system. Amplicon sequencing was performed on a HiSeq 2500 PE 250 (Illumina, Inc, USA).

Clean reads were filtered to get more accurate and reliable reads; this was achieved by splicing based on the overlap to get clean tags. The clean tag sequences obtained from each sample were combined, and the Usearch 10.0 standard UPARSE-OUT algorithm method was used for the cluster analysis of operational taxonomic units (OTUs) with a similarity of 97%. The similarity between the OTU sequence and the 16S sequence of known bacteria was compared to obtain the species classification information of OTU. Finally, the species and abundance of the microbiota in the samples were analyzed.

### 2.4 Measurement of SCFAs

SCFAs were detected by gas chromatography–mass spectrometry. One gram of stool and 10 mL of distilled water were put into a centrifuge tube. After being fully oscillated, a centrifuge was run at 12,000 rpm for 5 min. Next, 2 mL of the supernatant was diluted with 0.5 mL of 50% sulfuric acid and dissolved for 30 s. After that, 2 mL of ethyl ether was added, oscillated for 2 min, centrifuged for 10 min at 7,000 rpm, and 1 mL of the ethyl ether layer was used for gas chromatography-mass spectrometry analysis. The standard substances of acetic acid, propionic acid, and butyric acid were dissolved in ether as a standard solution. Helium was used as the carrier gas and the flow rate was set to be 1.18 mL/min. A temperature program was carried out as follows: start at 80°C for 1 min, at 20°C/min up to 180°C, then at 40°C/min up to 240°C, and maintained for 5 min. The inlet port temperature was 230°C, and the transmission line was 240°C, running for 12 min. The scan range was 20–200 *m*/*z* with the mode of SCAN and SIM. Monitored ions included acetate acid 43, 45, 60 *m*/*z*; propionate acid 29, 45, 73, 74 *m*/*z*; and butyrate acid 60, 73, 88 *m*/*z*. Ion source temperature was 200°C, and EI source bombardment energy was 70 eV.

### 2.5 RNA extraction and reverse transcription quantitative PCR

Total RNA was extracted from the frozen colon tissues using Trizol reagent (Wuhan Servicebio Technology Co., Ltd, Wuhan, China) and transcribed into cDNA using a cDNA synthesis kit (Thermo Fisher Scientific, New York, USA). The following primers were used: GPR43 F (5′-CGGACTTGCTGTTGTTGCT-3′) and GPR43 R (5′-TGCTCGGTTGAGTTCAGGTA-3′), β-actin F (5′-GTACCGCTGGGACCCTACAC-3′) and β-actin R (5′-AGCGTGGTGATGGCGTAGAA-3′). The amplification and detection of quantitative PCR products were performed with a 7500 real-time fluorescence quantitative PCR system (Corbett Company, Australia). The PCR was performed according to the following conditions: initial denaturation at 94°C for 31 s, denaturation at 94°C for 5 s, annealing at 60°C for 30 s, and extension at 60°C for 30 s, which was repeated for 40 cycles. The method of calculating GPR43 mRNA levels was the comparative 2^−ΔΔCt^.

### 2.6 Western blot analysis

Total protein was extracted from the frozen colon tissues by homogenization and quantified using the BCA protein quantitation assay (Fuzhou Watson Biotechnology Co., Ltd, Fuzhou, China). Equal amounts of protein (40 μg) and appropriate marker (3 μL) were loaded onto 10% sodium dodecyl sulfate-polyacrylamide gels, separated by electrophoresis, and then transferred to PVDF membranes. Subsequently, the membranes were incubated in 5% skim milk on a shaker at room temperature for 1 h, and then rinsed with Tris-buffered saline Tween (TBST) for three 5 min cycles. Membranes were then incubated overnight at 4°C with the primary antibody GPR43 (1:300; Proteintech, Chicago, USA). Membranes were washed with TBST as described above and incubated with the secondary antibody (Wuhan Servicebio Technology Co., Ltd, Wuhan, China) on a shaker at room temperature for 1 h, and then rinsed with TBST as before. Subsequently, equal doses of ECL reagent A and reagent B (Wuhan Servicebio Technology Co., Ltd, Wuhan, China) were mixed in a dark room. The PVDF membrane was put into the development box, and 200 µL of developer solution and reactor were added for 2 min. The membrane was placed in a dark box for exposure. After the exposure was completed, the exposure bands were photographed with an automatic gel imaging system (Liuyi Instrument Factory, Beijing, China). The gray value of the protein bands was measured, and the relative expression of the GPR43 receptor was calculated.

### 2.7 Measurement of GLP-1, PYY, and FBG levels in serum

Blood samples were centrifuged at 3,000 rpm for 15 min at 4°C, and the serum was collected and stored at −80°C for later analysis. Serum levels of GLP-1 and PYY were determined using an ELISA kit (Nanjing Jiancheng Bioengineering Institute, Jiangsu, China), and blood glucose concentration was measured by the glucose oxidase method with a reagent kit (Nanjing Jiancheng Bioengineering Institute, Jiangsu, China).

### 2.8 Statistical analysis

SPSS 20.0 software was used for data analysis, and data were expressed as mean ± standard deviation (SD). Body weight was analyzed using a repeated-measures analysis of variance, and FBG levels were analyzed by Student’s *t*-test. All other data or group differences were analyzed using one-way analysis of variance followed by LSD *post hoc* test or Dunnett’s T3 *post hoc* test. Statistical significance was defined as *P* < 0.05.

## 3 Results

### 3.1 Changes in body weight across intervention groups

**Figure 1:**
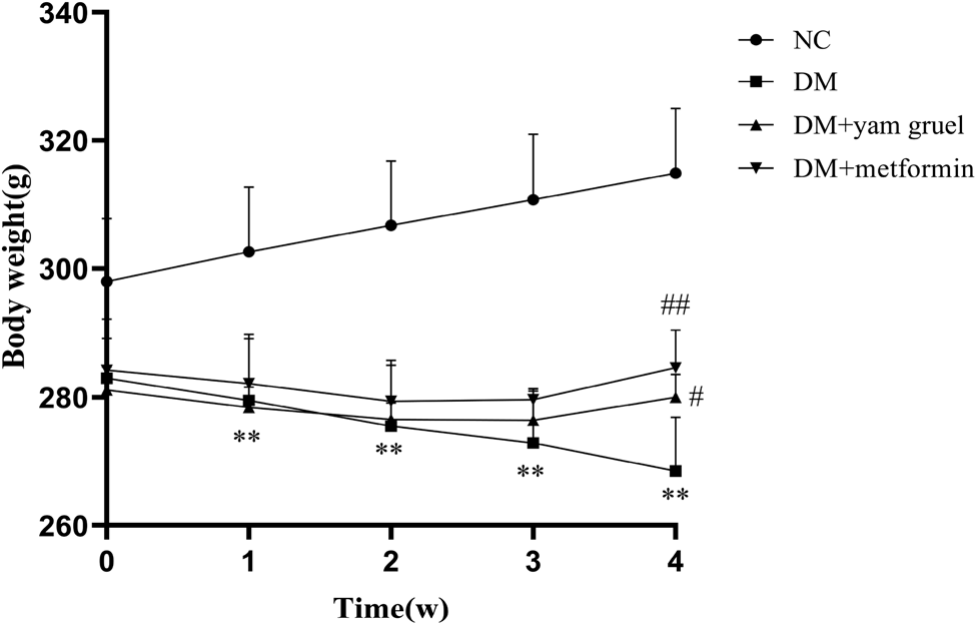
Changes in body weight during the 4-week intervention in different groups. Values are expressed as mean ± SD, *n* = 7 or 8. ^*^ *P* < 0.05 and ^**^ *P* < 0.01 vs NC group, ^#^ *P* < 0.05 and ^##^ *P* < 0.01 vs DM group.

During the intervention, the average body weight of the DM group was significantly lower than that of the NC group (*P* < 0.01). Additionally, after the administration of yam gruel or metformin, body weight was significantly increased at 4 weeks compared to the DM group (*P* < 0.05, Figure 1).

### 3.2 Effects of yam gruel on FBG

**Figure 2:**
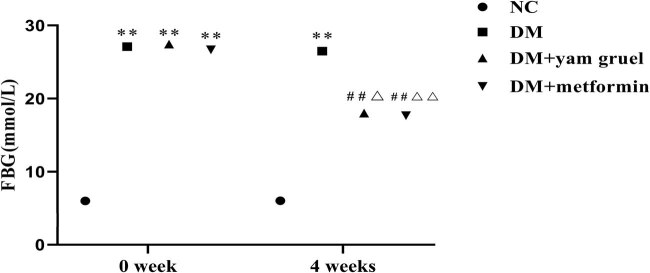
Effects of yam gruel on FBG. Values are expressed as mean ± SD, *n* = 7 or 8. ^*^ *P* < 0.05 and ^**^ *P* < 0.01 vs NC group, ^#^ *P* < 0.05 and ^##^ *P* < 0.01 vs DM group, ^Δ^ *P* < 0.05 and ^ΔΔ^ *P* < 0.01 vs 0 week.

To study the effect of yam gruel on the blood glucose levels of T2DM rats, FBG was measured before the intervention and at the end of a 4-week intervention. As shown in Figure 2, at the beginning of the intervention, there was no hyperglycemia in the NC group, while the high-sugar/high-fat diet combined with an intraperitoneal injection of STZ-treated rats showed different degrees of hyperglycemia. After 4 weeks of the intervention, compared with the NC group, FBG was significantly increased in the DM group (*P* < 0.01). Following the administration of yam gruel or metformin, the FBG levels were significantly lowered compared with the DM group (*P* < 0.01), but there were no significant differences between the two groups. In addition, intragroup comparison showed that the FBG levels of rats in the DM + yam gruel group and the DM + metformin group were significantly lower than those before intervention (*P* < 0.05).

### 3.3 Effects of yam gruel on the characteristics of gut microbiota

**Figure 3:**
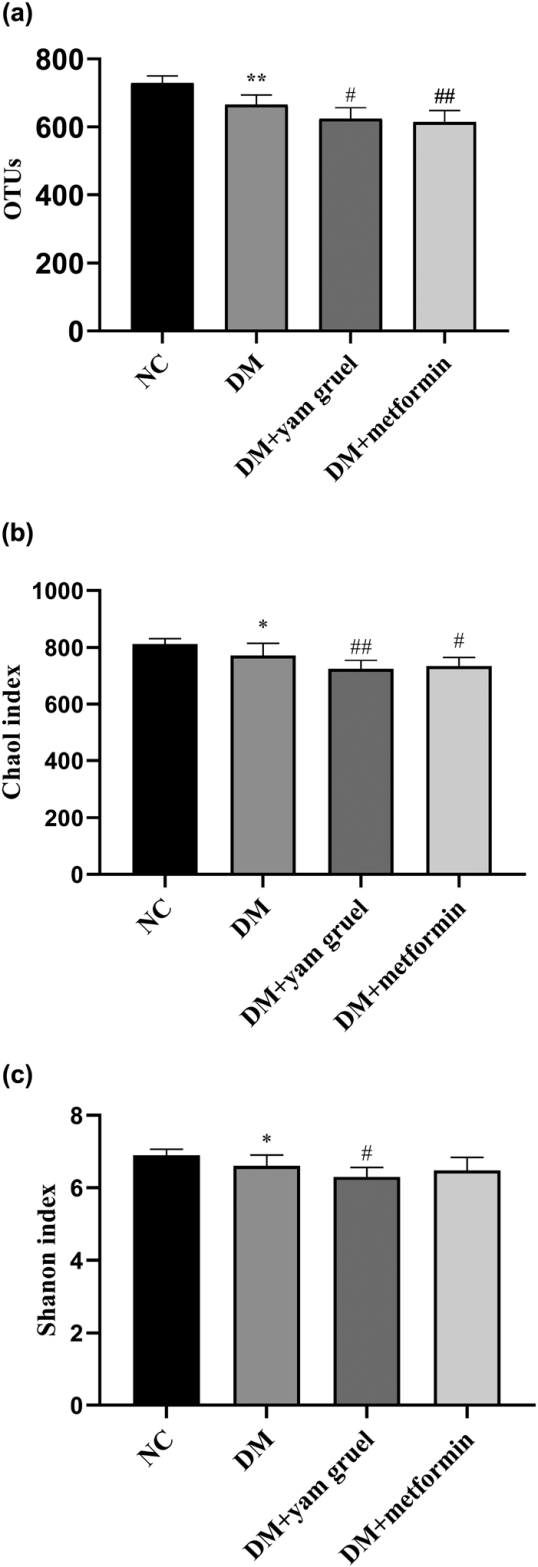
Effects of yam gruel on the characteristics of gut microbiota. (a) OTU numbers of each group. (b) Chao1 index of each group. (c) Shannon diversity index of each group. Values are expressed as mean ± SD, *n* = 7 or 8. ^*^ *P* < 0.05 and ^**^ *P* < 0.01 vs NC group, ^#^ *P* < 0.05 and ^##^ *P* < 0.01 vs DM group.

The characterization of gut microbiota was performed using OTU numbers, the Shannon diversity index, and the Chao1 index (Figure 3). Compared with that of the NC group, the numbers of OTU, Chao1 index, and Shannon index were significantly decreased in the DM group (*P* < 0.01). Additionally, supplementation of yam gruel could further reduce the above three indicators. However, only the numbers of OTUs were reduced significantly in the DM + metformin group compared with the DM group (*P* < 0.01).

### 3.4 Effects of yam gruel on the relative abundances of different bacteria in the gut microbiota

**Figure 4:**
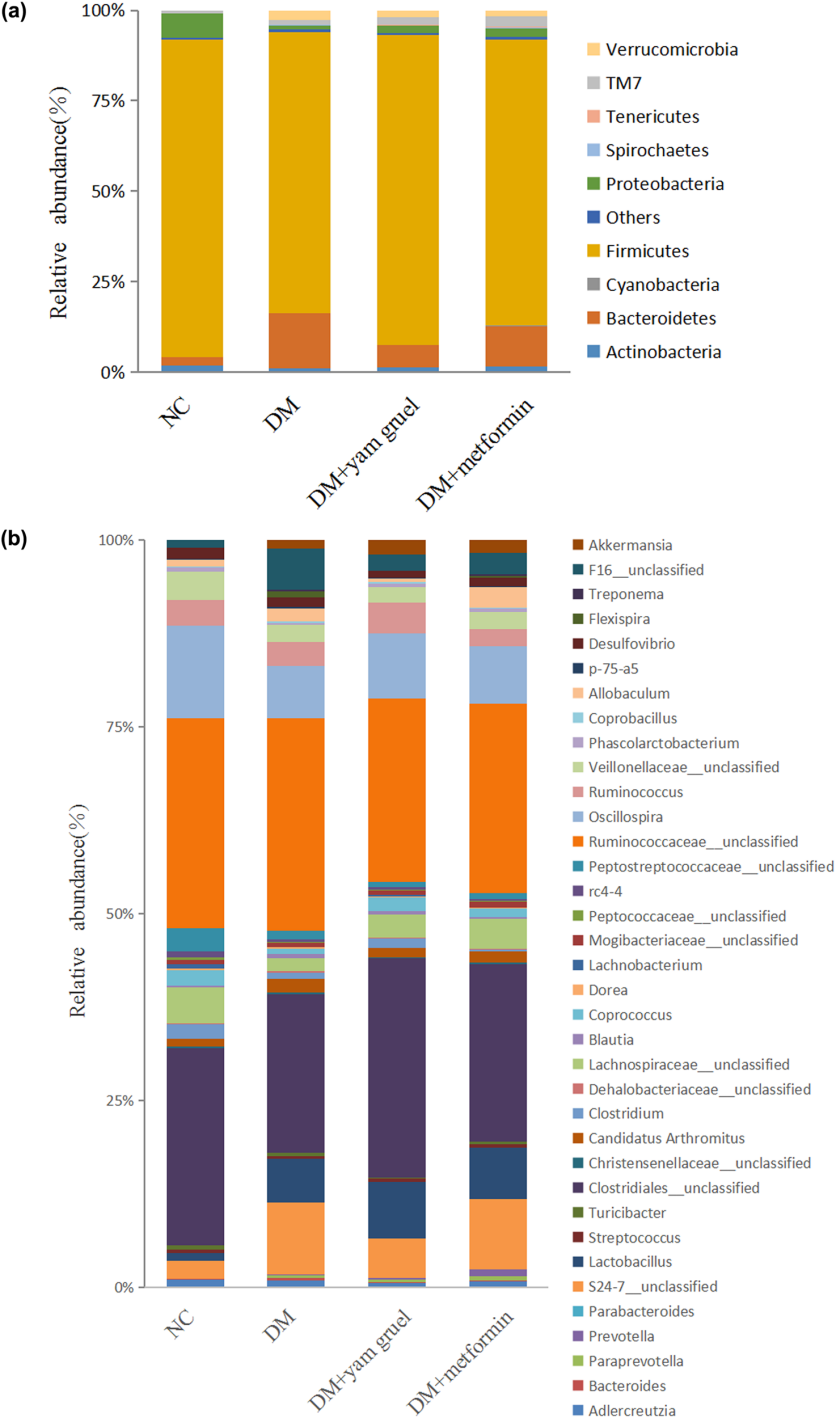
Effects of yam gruel on the relative abundances of gut microbiota at the (a) phylum and (b) genus taxonomic level in each group. Species that could not be annotated at the genus level were classified into the “unclassified” category.

At the phylum level (Figure 4a), the majority of the species in the gut microbiota were Firmicutes, Bacteroidetes, and Proteobacteria. There were some differences between the groups. Compared with the NC group, the DM group had a significantly decreased abundance of Firmicutes and an increased abundance of Bacteroidetes (*P* < 0.05). However, the intervention of yam gruel or metformin increased the abundance of Firmicutes and decreased the abundance of Bacteroidetes in T2DM rats (*P* < 0.05). At the genus level (Figure 4b), there was a reduced abundance of SCFA-producing bacteria (such as Streptococcus, Oscillospira, and Phascolarctobacterium) in the DM group compared to the NC group (*P* < 0.05). Furthermore, rats in the DM group had a greater abundance of Bacteroides (*P* < 0.05). Yam gruel or metformin supplementation significantly elevated the levels of Streptococcus, Oscillospira, and Phascolarctobacterium and decreased the Bacteroides level compared with the DM group (*P* < 0.05).

### 3.5 Effects of yam gruel on SCFAs

**Figure 5:**
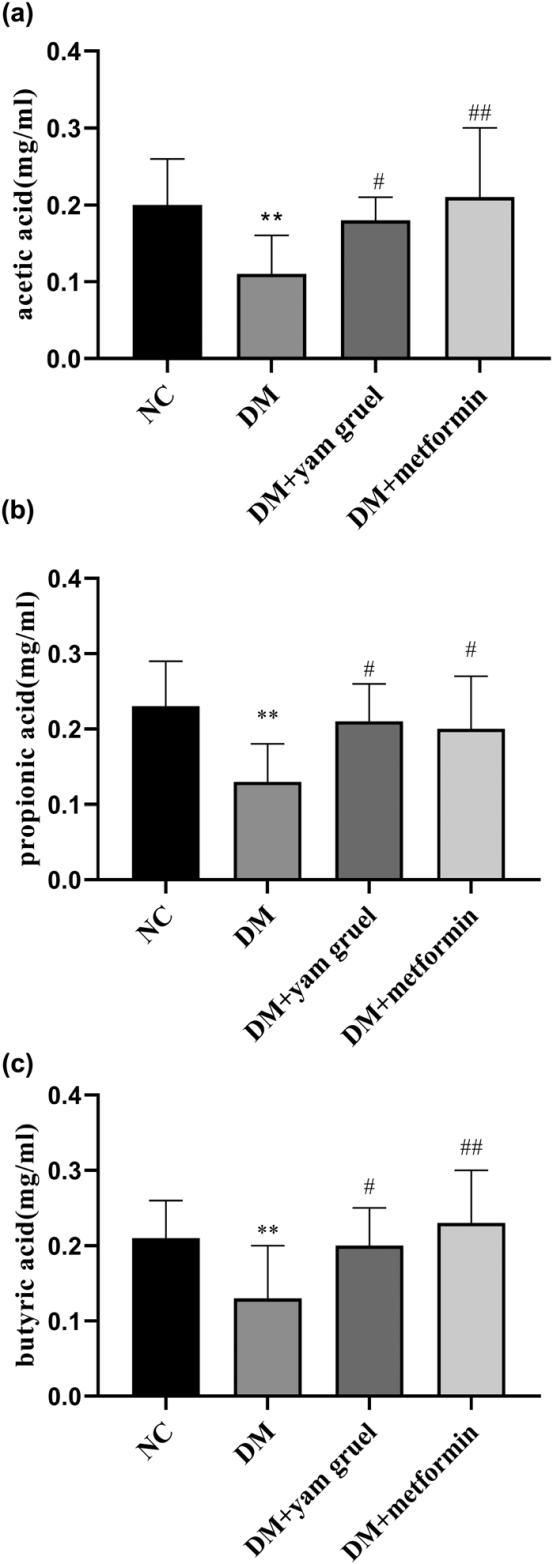
Effects of yam gruel on SCFAs. (a) Acetic acid level of each group. (b) Propionic acid level of each group. (c) Butyric acid level of each group. Values are expressed as mean ± SD, *n* = 7 or 8. ^*^ *P* < 0.05 and ^**^ *P* < 0.01 vs NC group, ^#^ *P* < 0.05 and ^##^ *P* < 0.01 vs DM group.

SCFAs (including acetic acid, propionic acid, and butyric acid) were measured as shown in Figure 5. Comparing the concentration of acetic acid, propionic acid, and butyric acid between the NC group and the DM group, all had a lower level in the DM group (*P* < 0.05). Additionally, there was an increased concentration of acetic acid, propionic acid, and butyric acid after 4 weeks of supplementation with yam gruel or metformin in T2DM rats.

### 3.6 Effects of yam gruel on the GPR43 receptor expression in colon tissues

**Figure 6:**
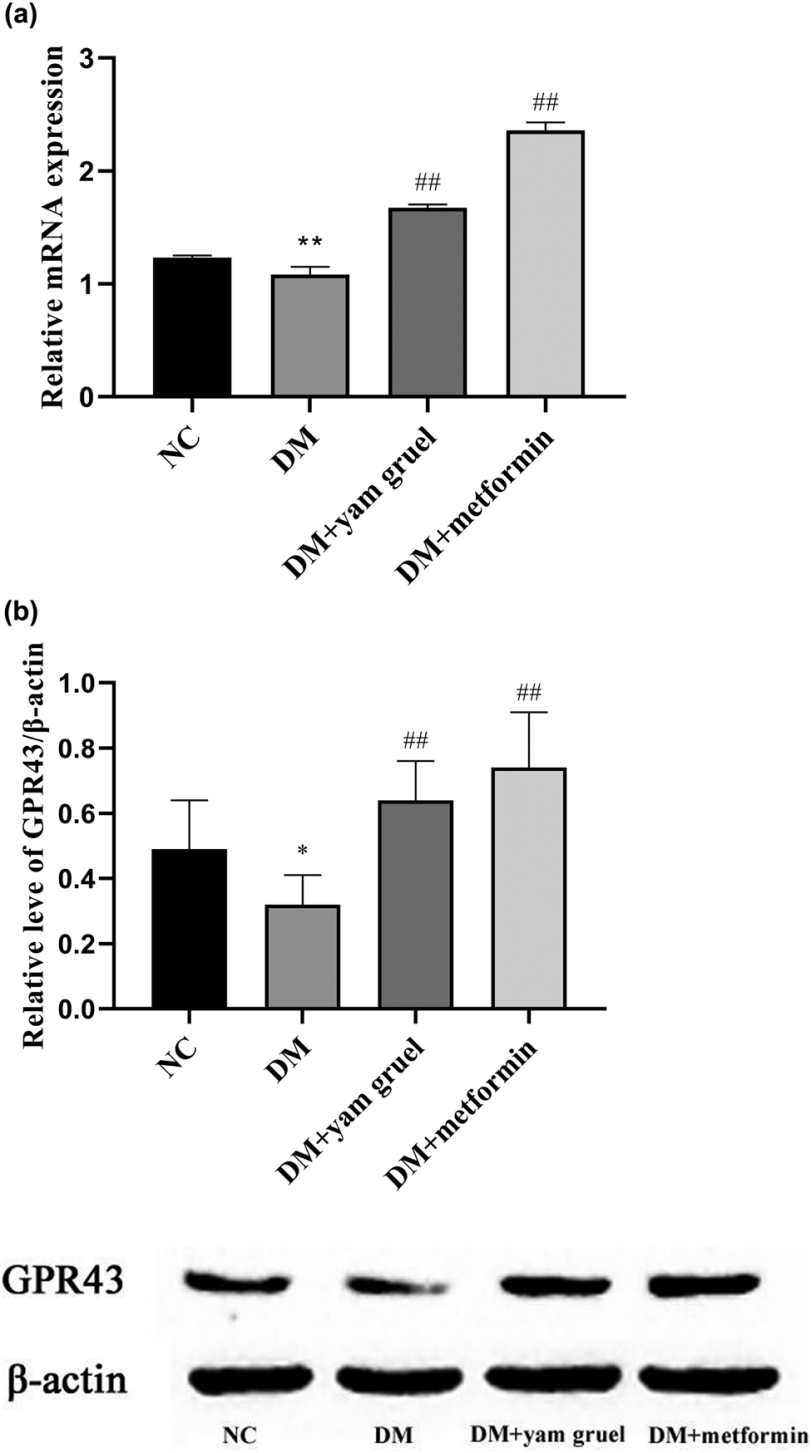
Effects of yam gruel on the GPR43 receptor expression in colon tissues. (a) mRNA levels of GPR43 of each group. (b) Protein levels of GPR43 of each group. Values are expressed as mean ± SD. ^*^ *P* < 0.05 and ^**^ *P* < 0.01 vs NC group, ^#^ *P* < 0.05 and ^##^ *P* < 0.01 vs DM group.

As shown in Figure 6, compared with the NC group, both gene and protein levels of the GPR43 receptor were significantly reduced in the DM group (*P* < 0.01). Additionally, the expression of the GPR43 receptor was significantly enhanced in the DM + yam gruel group and in the DM + metformin group (*P* < 0.01).

### 3.7 Effects of yam gruel on the levels of GLP-1 and PYY in serum

**Figure 7:**
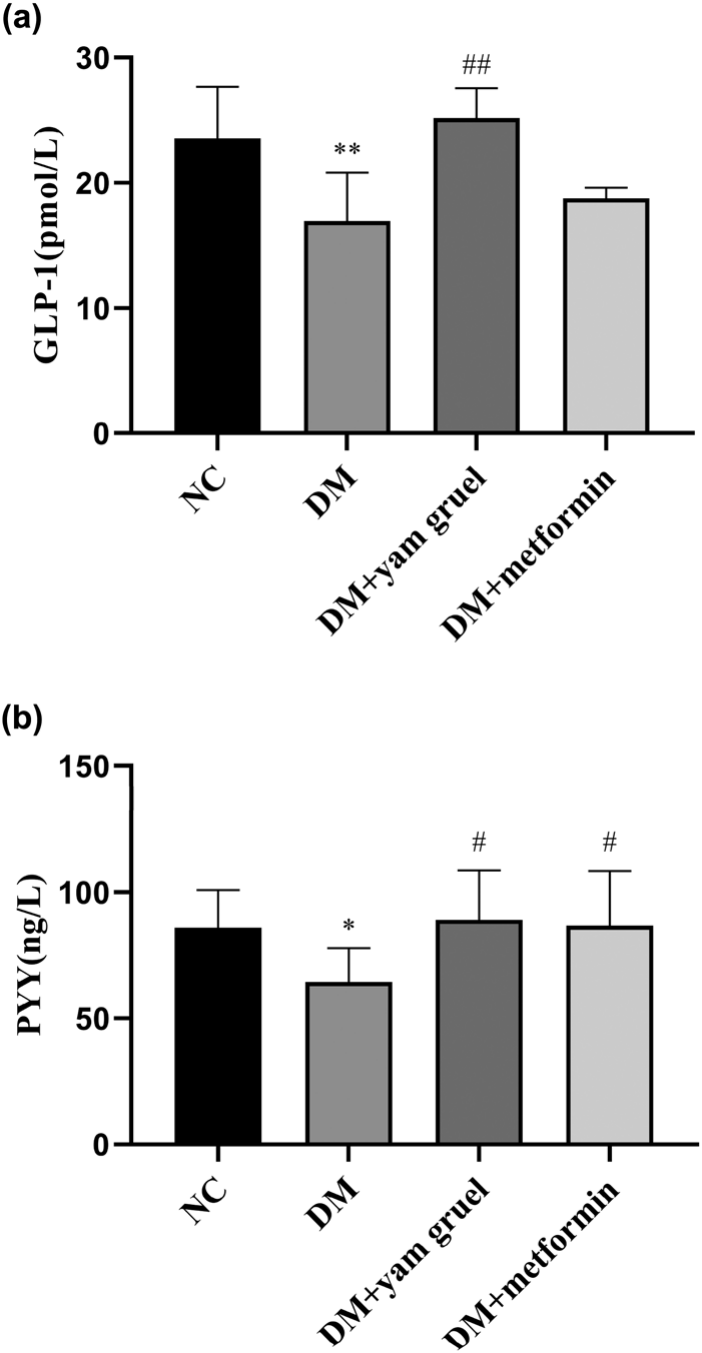
Effects of yam gruel on the levels of GLP-1 and PYY in serum. (a) GLP-1 levels of each group. (b) PYY levels of each group. Values are expressed as mean ± SD, *n* = 7 or 8. ^*^ *P* < 0.05 and ^**^ *P* < 0.01 vs NC group, ^#^ *P* < 0.05 and ^##^ *P* < 0.01 vs DM group.

GLP-1 and PYY were measured as shown in Figure 7. Comparing the levels of GLP-1 and PYY in the NC group and the DM group, both were lowered in the DM group (*P* < 0.05). There was also an increased level of GLP-1 and PYY after the supplementation of yam gruel in T2DM rats. On the contrary, only the PYY levels displayed significant differences between the DM group and the DM + metformin group.

## 4 Discussion

In this study, we investigated the antidiabetic effect of yam gruel in T2DM rats and analyzed the potential hypoglycemic mechanism. The results demonstrated that yam gruel could significantly reduce the diversity of gut microbiota, promote the growth of probiotic bacteria (especially SCFA-producing bacteria) in T2DM rats, and enhance the expression of GPR43 receptor in colon tissues to increase the content of GLP-1 and PYY in serum and play a hypoglycemic role. In addition, metformin (the preferred oral drug for lowering hyperglycemia [18]) was selected as the positive control drug in the present study. We found that the levels of FBG were significantly lower in T2DM rats treated with yam gruel or metformin and that there were no significant differences between the two groups, which suggests that the hypoglycemic effect of yam gruel was similar to metformin. In summary, dietary supplementation of yam gruel might be useful in improving fasting hyperglycemia, which provides a novel therapeutic option for the prevention or treatment of T2DM.

T2DM is a chronic metabolic disease characterized by fasting hyperglycemia, which is affected by multiple factors such as genetic, gut microbiota, lifestyle, and dietary patterns [19]. With advancements in research, the relationship between gut microbiota and T2DM has been further clarified. Zhao et al. [8] reported that there was a moderate-intensity imbalance of gut microbiota in T2DM patients, which was mainly manifested as the decrease in probiotic bacteria and the increase in pathogenic bacteria. Additionally, SCFAs as the primary metabolite of intestinal probiotic have been proposed to be closely related to the level of FBG. For example, increasing the content of SCFAs is conducive to improve insulin resistance and stable maintenance of blood glucose [11]. According to a clinical trial in 2017, T2DM patients treated with multiple strains of probiotics for 12 weeks demonstrated significantly improved insulin resistance [20]. Zhao et al. [12] reported that the enhanced abundance of Phascolarctobacterium and other probiotic bacteria could reduce the release of inflammatory factors and suppress hepatic gluconeogenesis, resulting in a lower level of FBG. Our data showed that the intervention of yam gruel could promote the growth of intestinal probiotics such as Streptococcus, Oscillospira, and Phascolarctobacterium, increase the content of SCFAs, and significantly reduce the level of FBG in T2DM rats. We speculated that this might be related to the composition of yam gruel. Yam gruel consists only of raw Chinese yam, which is regarded as a natural, hypoglycemic crop that has been widely used in the prevention and treatment of diabetes in ancient China [21].

On the other hand, modern medicine postulates that Chinese yam is rich in resistant starch, dietary fiber, polysaccharides, and other components that can regulate intestinal probiotic bacteria to play a role in lowering blood glucose [22]. Lyte et al. [23] found that the number of probiotics significantly increased and the level of FBG decreased after treatment with resistant starch for 6 weeks in T2DM mice. Moreover, the content of dietary fiber was negatively correlated with blood glucose [24]. In particular, the increase in water-soluble dietary fiber can be fermented by intestinal probiotic bacteria to produce SCFAs. The increase in SCFAs can subsequently reduce the pH value in the intestinal tract, enhancing the acidic environment of the intestinal tract and promoting the growth of probiotics so as to achieve the goal of reducing blood glucose.

GPR43 is an essential member of the orphan receptor in the G protein-coupled receptor superfamily and is also the specific receptor of various SCFAs. Kim [25] proposed that the content of SCFAs was positively correlated with the expression of GPR43 receptor and that a high concentration of SCFAs could upregulate the level of gene and protein expression of GPR43 receptor. This was consistent with the views of Liang et al. [26]. GPR43 receptor has been found to be closely related to the development of T2DM [27]. Activation of GPR43 receptor could increase Ca^2+^ in intestinal L cells, further promoting the secretion of GLP-1 and PYY so as to lower the level of FBG. It has been demonstrated that mice with GPR43 gene knockout presented an obese phenotype, excessive accumulation of fat, and predisposition to obstacles of insulin signal transmission, thus inducing insulin resistance [28]. According to an animal experiment, supplementation with probiotic bacteria upregulated the expression of GPR43 receptor, repaired glucose intolerance, and reduced the level of blood glucose in T2DM rats [29]. Furthermore, the mechanism of action may be related to the improvement of the concentration of SCFAs. In our study, yam gruel treatment enhanced the expression of GPR43 receptor in colon tissues, which may be due to the increased abundance of probiotic bacteria. The increased probiotic abundance stimulating the content of SCFAs in turn may have facilitated the upregulation of GPR43 receptor.

GLP-1 and PYY are gastrointestinal peptide hormones secreted by L cells at the end of the small intestine and the colon. They play an important role in regulating appetite, reducing body mass, and maintaining blood glucose stable [30]. Previous studies have shown that in the pathological state of T2DM, intestinal L cells display dysfunctions of secretion and GLP-1 and PYY levels are significantly lower than usual [31]. Interestingly, increases in the population of GLP-1 and PYY can significantly ameliorate the insulin resistance and glucose homeostasis of T2DM [32]. For one, GLP-1 can help to promote the proliferation and regeneration of β-cells, prevent their apoptosis, and stimulate the secretion of insulin. In addition, it also inhibits the secretion of glucagon by α-cells in a glucose-dependent mode. This not only has an effect on the reduction of FBG but can also effectively prevent the occurrence of hypoglycemia [33]. Additionally, GLP-1 is expected to exert an impact on appetite regulation and food absorption to control body weight. Compared with GLP-1, PYY reduced the level of FBG mainly by delaying the emptying of gastric content, enhancing satiety, reducing food intake, and controlling body weight to improve insulin sensitivity [34]. Our data showed that the content of GLP-1 and PYY in serum was reduced notably in T2DM rats. Dietary supplementation of yam gruel, on the other hand, elevated serum GLP-1 and PYY levels. This was mainly due to the active ingredient of yam in yam gruel. Resistant starch and dietary fiber in Chinese yam can increase the content of SCFAs [35], which may help to upregulate the expression of GPR43 receptor. Psichas et al. [36] confirmed that SCFAs significantly promoted GLP-1 and PYY secretion through an animal experiment. SCFAs are considered the most effective ligand for GPR43 receptor. The binding of GPR43 receptor and SCFAs can likely stimulate intestinal L cells to secrete GLP-1, PYY, and other gastrointestinal peptide hormones so as to exert an antidiabetic function [13].

In conclusion, our study indicated that dietary supplementation with yam gruel could promote the growth of probiotic bacteria and SCFAs in T2DM rats, thus upregulating the expression of GPR43 receptor in colon tissues and increasing serum GLP-1 and PYY levels. All of these factors may work together to trigger yam gruel’s antidiabetic effects. These findings may be valuable for the prevention and treatment of diabetes with safer, natural compounds.
